# Clinical and Pathogenetic Notes

**Published:** 1884-10

**Authors:** E. W. Berridge

**Affiliations:** London


### CLINICAL BUREAU.

---

### CLINICAL AND PATHOGENETIC NOTES.

E. W. Berridge, M. D., London.

(1.) *Mephites.*—Pain in left hypochondrium as from flatus there; worse by moving left arm across body. The part feels bruised on touch. One dose of *Mephites putorius*$^{200}$ removed the pain within an hour and it did not return. (See C. Lippe's *Repertory*, p. 113.)

(2.) *Calcarea.*—Mrs. J. reports that *Calcarea*$^{3}$, half a drop twice a day, caused her baby to urinate six or seven times an hour, passing a good deal each time. This ceased soon after leaving off the medicine.

(3.) *Cinchona.*—Constant choking; every day, between 6 and 7 P. M., short breath and loud wheezing, making the choking worse; must sit up and sip water. Tight, stuffy cough, with very little sputa. Pain in spine, like a weight there; worse by lifting; sensation of fullness in abdomen while eating; all her clothes feel tight; constant tiredness; must lie down, else she has such palpitation that she cannot breathe; head dragged backward. *Cinchona*$^{cm}$ (Fincke) quickly cured.

(4.) *Pulsatilla.*—For three days cannot retain food; it comes up hours afterward just as swallowed; vomiting and purging at the same time. During retching, cold sweat; constant shivering; constant bitter taste—even the saliva is bitter. *Pulsatilla* (high potency) cured.

(5.) *Nitric acid.*—Great constipation; piles, with bearing down on standing; bread disagrees. *Nitric acid*$^{mm}$ (Fincke) cured "like magic."

(6.) *Sepia.*—Dull, aching pain all round body and feeling of something hard boring through liver; spasms in liver, which catch the breath; fullness and bursting, relieved by urination. *Sepia*$^{cm}$ (Fincke) cured.

(7.) *Aurum.*—May 23d, 1881. Dragging aching at back of eyes on reading, sewing, or writing—relieved by closing eyes; constant smarting in eyes; lachrymation; lids red with itching; threatening of styes. *Aurum*$^{cm}$ (Fincke).

June 3d. Much improved till this morning. Now there is smarting like salt in right eye; streaming of acrid water from right eye and right nostril, excoriating cheek; photophobia; redness of right eye; bright spots before eyes by gaslight; feeling of squinting inward after removing spectacles; pain in eyes better by blowing nose. A repetition of *Aurum*$^{cm}$ (Fincke) cured. This verifies Hering's *Guiding Symptoms.*

(8.) *Lachesis.*—For six weeks, on left calf, a hard red patch with scabs on it; it is tender and discharges a thin, slightly yellow fluid from under the scabs; it itches, especially in cold weather. In the right popliteal space is a similar appearance, but not so bad, and of more recent occurrence. One dose of *Lachesis*$^{cm}$ (F. C.) cured. The right leg was the first to get well, in accordance with the law of *Inverse Directions.*

(9.) *Chamomilla.*—Miss S. informs me that she has cured in herself bitter taste in mouth—removed by washing mouth out, on bitter vomiting, with a high potency of *Chamomilla.*

(10.) *Chelidonium.*—Mr. C. took *Chelidonium majus*$^{200}$ three times a day for some eye symptoms. It improved the old symptoms, but caused pain, as from an insect in left eye, at times throughout the day, with constant *profuse* lachrymation. Two days after leaving it off the pain ceased and the lachrymation subsided to its former extent.

(11.) *Phosphorus.*—During stool, shooting pain, commencing in rectum and going up spine to occiput very often, for three or four years, in an old lady of sixty-three. *Phosphorus*$^{mm}$ (Fincke) cured in two doses.

(12.) *Calcarea.*—For two months a hard lump in left upper eyelid, preventing the full opening of the eye; it has been increasing for the last two or three weeks; for about a week, aching in the lump on looking long at anything. For a month, *feet feel as if she had damp stockings on.* For a week, back of hands cold to touch and numb. One dose of *Calcarea carb.* $^{10mm}$ (Swan) cured. The aching ceased before the lump disappeared.

(13.) *Sulphur.*—The same patient; enlarged vein in right calf, noticed for last six days, with aching, tingling, and burning in it. One dose of *Sulphur*$^{cm}$ (F. C.) cured it without the use of any mechanical aid.

(14.) *Medorrhinum.*—Mr. J. reports that when he had gonorrhœa last year he felt better in health than before, less constipated and free from indigestion. (Compare provings of *Medorrhinum.*)

(15.) *Lycopod.*—Miss H. took several doses of *Lycopod.*$^{cm}$ (Fincke) for some chronic symptoms. It caused shooting pains in chest, darting from one part of chest to another, worse on deep inspiration.

(16.) *Sulphur.*—Miss S., æt. about forty. Had pleurisy at age of twenty-two. Ever since then has been subject to the following symptoms: Pain in left lower thorax, back and front, as if something fell forward, on turning over on to right side when lying; if she continues to lie on right side it feels as if it pressed against front lower chest; if she still continues in that position there is also heat in the part affected; the pressure causes dyspnœa when lying on right side. It has been bad for last six months. Nothing has relieved it, not even *Sulphur* 2m (Jenichen) and cm (Fincke) which I had given her for other symptoms, not knowing of these. On September 12th, 1874, I gave her one dose of *Sulphur* mm (Bœricke) at 9 P. M. The same evening she improved. When I came next morning she had the following new symptoms: Great feeling of weakness, as after a severe illness; dreamed that she was dying, that all was getting dark, and that she called out in a great fright; then she awoke, feeling weaker and worse than before; the weakness lasted badly an hour or two, and she felt more or less weak all day.

October 13th. The pain in chest has steadily improved, with one very severe aggravation, and yesterday, for the first time in her life, it was quite gone.

Subsequently the symptoms returned, and she took Swan's 10mm, and afterward 20mm (both made with a continuous stream of water from first to last). The 10mm, she said, did more good than the mm, and the 20mm the most good of all. Once *Sulphur* 20mm relieved greatly, after an aggravation, when the mm had failed to act. She required a repetition of the medicine from time to time, but at length recovered so far that she never complained of the pains.

(17.) *Tabacum.*—Mr. Thomas D., August 23d, 1878. Yesterday, about 11 A. M., dim sight; could only see the last letter when writing; this lasted forty-five minutes; as it went off numbness of tongue ensued, especially at the back part, also numbness of hands, worse in fingers; this lasted two hours, going off gradually; after numbness had gone there was a tingling pain in forehead as after a severe slap there, worse from noise; this has lasted till the present time. This morning there is shooting headache from over left eye backward half way to occiput; tongue trembles when protruded. Has had several attacks in the last fifteen years. He began to smoke tobacco at age of ten. When he used to smoke much he had attacks every two or three months; then he ceased almost entirely; the attacks ceased also. Has smoked more lately, and the attacks have returned, though they had been absent four years. There has been no worry or excessive heat to account for this attack. I gave him one dose of *Tabacum*$^{1cm}$ (Fincke). Next day had a new symptom, numbness of right calf, that recurred at intervals for several days. No further report.

(18.) *Lycopodium.*—Cutting pains from left scapula to chest, catching the breath; feels very ill and exhausted, with intolerable thirst at 4 P. M.; one dose of *Lycopod.*$^{43m}$ (Fincke) cured.

(19.) *Sulphur.*—For four days swelling and redness of inner side of right foot and right inner malleolus; the part is very tender, as is also right outer malleolus; sharp darting pain from right inner malleolus, going up to knee, and down along instep toward toes; worse by standing on it with foot flat; can stand a little on the toes. One dose of *Sulphur*$^{mm}$ (F. C.) cured promptly.

(20.) *Calcarea.*—Miss G., æt. fifty, took repeated doses of *Calcar. carb.*$^{3cm}$ (Swan) for some chronic symptoms; it produced the following new symptoms, which ceased after leaving off the medicine: burning feeling in soles, which were not hot to touch, with feeling of swelling there, preventing sleep.

(21.) *Eucalyptus.*—Tightness across bridge of nose on first lying down in bed was cured by *Eucalyptus*$^{30}$.

(22.) *Thea.*—Mr. N., æt. fifty, reports that tea causes in him trembling of whole body, voice trembles when singing, and a slight imperfection in articulation when speaking, a sort of nervous stuttering; also diarrhœa preceded by griping.

(23.) *Pulsatilla.*—Mrs. D., mother of several children, and nearly four years past the change of life, took a high potency of *Pulsatilla* for some chronic symptoms; it produced the following new symptoms: Feeling as if the milk were running into breasts, which feel distended. This patient was re-vaccinated eighteen years ago and was very ill after it, with abscesses extending down to elbow.

(24.) *Arsenicum.*—Mr. H. V. D., æt. twenty-one, took several doses of a high potency of *Arsenicum* for symptoms of phthisis. It caused the following: Involuntary urinations waking him at 3 A. M. precisely; he had drank but little, and had never had nocturnal emissions since childhood.

(25.) *Thea.*—Miss I. reports that a cup of tea, which she had not taken for years, brought on urticaria.

(26.) *Aloes.*—Mrs. S., æt. sixty-three, had pains in rectum, worse when walking; once the solid stool dropped out involuntarily. *Aloes*$^{cm}$ (Fincke) cured.

(27.) *Vaccination.*—Mr. J. was re-vaccinated ten years ago and had a very bad arm; since then has been much more free from gout.

(28.) *Phosphorus.*—Miss —— took several doses of *Phosph.*$^{cm}$

(Fincke) for neuralgia of the right side of face ; after taking it, when the paroxysms came on, the right side of mouth filled with *cold saliva*, which was not the case before. (Compare Bœnninghausen's *Repertory*.)

(29.) *Pulsatilla.*—November 23d, 1883, Mrs. B. had cough with expectoration after rising in morning ; cough causes involuntary urination, and retching sometimes to vomiting; *the retching causes pains across sacral regions.* One dose of *Pulsatilla*$^{cm}$ (F. C.) cured.

